# Mathematical Model for Growth and Rifampicin-Dependent Killing Kinetics of *Escherichia coli* Cells

**DOI:** 10.1021/acsomega.3c05233

**Published:** 2023-10-05

**Authors:** Meltem Elitas, Guleser Kalayci Demir, Sumeyra Vural Kaymaz

**Affiliations:** † Faculty of Engineering and Natural Sciences, Sabanci University, Istanbul 34956, Turkiye; ‡ Faculty of Engineering, Department of Electrical and Electronics Engineering, Dokuz Eylul University, Izmir 35397, Turkey

## Abstract

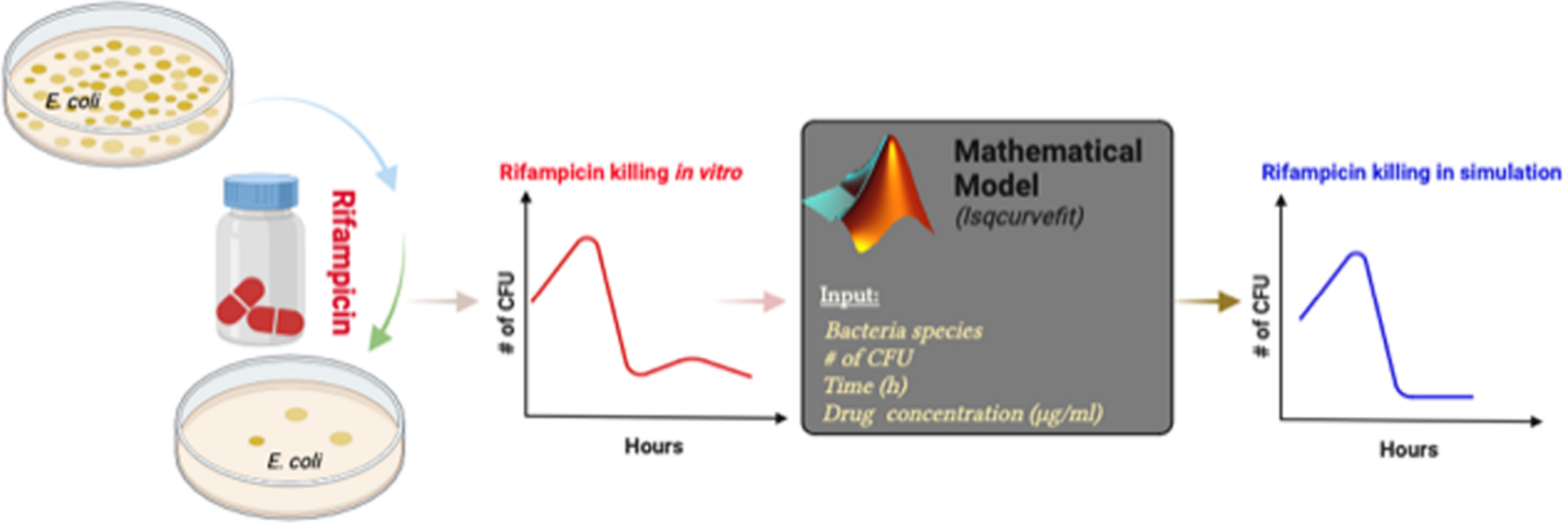

Antibiotic resistance is a global health threat. We urgently need better strategies to improve antibiotic use to combat antibiotic resistance. Currently, there are a limited number of antibiotics in the treatment repertoire of existing bacterial infections. Among them, rifampicin is a broad-spectrum antibiotic against various bacterial pathogens. However, during rifampicin exposure, the appearance of persisters or resisters decreases its efficacy. Hence, to benefit more from rifampicin, its current standard dosage might be reconsidered and explored using both computational tools and experimental or clinical studies. In this study, we present the mathematical relationship between the concentration of rifampicin and the growth and killing kinetics of *Escherichia coli* cells. We generated time-killing curves of *E. coli* cells in the presence of 4, 16, and 32 μg/mL rifampicin exposures. We specifically focused on the oscillations with decreasing amplitude over time in the growth and killing kinetics of rifampicin-exposed *E. coli* cells. We propose the solution form of a second-order linear differential equation for a damped oscillator to represent the mathematical relationship. We applied a nonlinear curve fitting solver to time-killing curve data to obtain the model parameters. The results show a high fitting accuracy.

## Introduction

Antibiotic resistance is a global health threat; particularly, it emerges during wars, mass migrations, and pandemic conditions. Under these circumstances, to combat infections, emergency strategies need to be applied.^1^ These strategies can be discovery of an antibiotic,^2,3^ invention of an alternative to antibiotics,^4−8^ or reformulation of the administration of current antibiotics by a better understanding of bacterial behavior.^9,10^ Currently, a limited number of antibiotics are in the treatment repertoire existing for bacterial infections.^11^ Among them, rifampicin is one of the essentials that has been used for the treatment of various mycobacterial and Gram-positive bacterial infections.^14−22^ In the literature, several clinical and computational studies have been conducted to shorten therapy time, increase effectiveness, and reduce the negative side effects and financial costs of rifampicin treatments.^23,24^ Several lines of evidence suggest that under conditions of tolerability and safety, intensified regimens incorporating elevated dosages of rifampicin may shorten the treatment duration or contribute to the management of infections linked to high mortality rates. Nevertheless, a consensus remains elusive regarding assessing pharmacokinetic parameters, efficacy clarification, and compound toxicity. In this study, we propose a mathematical model between the concentration of rifampicin and the growth and killing kinetics of *Escherichia coli* (*E. coli*) cells. We particularly focus on modeling the fluctuations in the antibiotic responses of cells and propose to use the solution of the damped oscillator equation to obtain the mathematical relationship between the rifampicin concentrations and the growth and killing kinetics of *E. coli* cells. Along this line, upon obtaining time-killing curves of *E. coli* cells in the presence of 4, 16, and 32 μg/mL rifampicin exposures, we use the lsqcurvefit function in MATLAB to find model parameters of damped oscillatory behavior of cells. To the best of our knowledge, this is the first study that has modeled the fluctuations in the growth and killing kinetics of *E. coli* cells in the presence of rifampicin with high accuracy. We believe that our results might contribute to elaborate interrogations on better understanding of initial antibiotic killing phases of antibiotic treatments in the context of antibiotic resistance.

## Literature

Rifampicin has been an important medicine since it was discovered in 1965. It is still on the World Health Organization’s List of Essential Medicines. Rifampicin inhibits bacterial DNA-dependent RNA polymerase and suppresses RNA synthesis to kill bacteria.^12,13^ It is widely used for the treatment of tuberculosis,^14^ leprosy,^15^ acute bacterial meningitis,^16−18^ pneumonia,^19,20^ and biofilm-related infections.^21,22^ In pursuit of enhanced therapeutic outcomes, several clinical and computational studies have been conducted to shorten the therapy time, increase its effectiveness, and reduce the negative side effects and financial costs of rifampicin.^23,24^ In the context of antibiotic administration, generally, lower cure rates can be attributed to the increased emergence of resistance within infections.^25^ Nonetheless, when tolerability and safety of the elevated dosages of rifampicin are achieved, it might contribute to the management of infections with high mortality rates.

Developing new computational models might help guide experiments and clinical tests to determine the optimal dosage for achieving favorable cure rates, reduced relapse rates, minimal toxicity, and lower mortality rates. Mostly, to obtain the optimal rifampicin dose that can increase the speed of the sterilizing effect, several studies have evaluated the pharmacokinetic–pharmacodynamic index of rifampicin using *in vitro* models of tuberculosis^23,29,30^ and in patients.^31−33^ The landmark study by Weinstein and Zaman reported the evolution of rifampin resistance in *E. coli* and *Mycobacterium smegmatis* due to substandard drugs. They demonstrated substandard drugs that contain degraded active pharmaceutical ingredients (rifampin quinone) select for gene alterations that confer resistance to standard drugs.^34−36^ Along the same line, Regoes and co-workers focused on developing mathematical models to describe the relationship between the bacterial net growth rates and the concentration of antibiotics.^37^ They presented a pharmacodynamic function based on a Hill function and exhibited that pharmacodynamic parameters might influence the microbiological efficacy of treatment. The descriptive model developed by Guerillot and co-workers considers the lag phase, the initial number of bacteria, the limit of effectiveness, and the bactericidal rate of antimicrobial agents.^38^ Their model was applied to compare the time-killing curves of amoxicillin, cephalothin, nalidixic acid, pefloxacin, and ofloxacin against two *E. coli* strains.

As mentioned above, several studies have provided evidence of a dose-dependent clinical response to rifampicin.^23,26−28^ Despite these considerations, there has been very limited systematic study exploring the quantitative relationship between the concentration of rifampicin and the growth and killing kinetics of *E. coli* cells.^34,37,39^

## Methodology

### Bacterial Strain and Growth Curves

The strain used in this study is American Type Culture Collection (ATCC) 10536 derived from *E. coli* K-12. Bacteria from the glycerol stocks were inoculated into 2 mL of Miller’s Luria–Bertani Broth (LB) and incubated at 37 °C by shaking at 200 rpm for ∼16 h. A spectrophotometer (BIOCHROM, WPA Biowave II ultraviolet/visible (UV/vis), U.K.) was used to determine the turbidity of cultures by measuring their optical densities (absorbance at 600 nm). To obtain growth kinetics, *E. coli* cells were grown to OD_600_ of 0.5–1 and then diluted to OD_600_ of 0.05 in a fresh LB medium. To obtain the growth kinetics of the antibiotic-treated bacterial cultures, 200 μL of bacterial cultures was transferred into the microplate wells with the following rifampicin concentrations: 4, 16, 32 μg/mL. Following the preparation of microplate wells, the plates were sealed and positioned within a microplate reader (TECAN Multimode Microplate Reader) for further analysis. During the measurements, the plates were rotated at 200 rpm with 20 min intervals at 37 °C for 4500 min.

### Antimicrobial Agent

Rifampicin molecule (Sigma-Aldrich, catalog no. R-120) was carefully weighted to obtain 0.1 g and dissolved in dimethyl sulfoxide (DMSO, PanReac Applichem, catalog no. P100C16), resulting in a concentration of 10 mg/mL. Subsequently, this prepared stock solution was employed to obtain the subsequent rifampicin concentrations: 4, 16, and 32 μg/mL. In our experiment, the doses of 32 μg/mL were negligible compared to toxic levels of rifampicin.^40^ Hence, adverse reactions and toxicity were not considered, as they were beyond the bounds of this study.

### Colony-Forming Unit (CFU) Assay

Serial 10-fold dilutions of bacteria culture were made using 100 μL of bacteria culture and 900 μL of 1× phosphate-buffered saline (PBS)—0.025% Tween 20 solution in 5 mL polypropylene tubes. Previous studies have demonstrated that Tween 20 did not significantly inhibit the growth of *E. coli* cells at concentrations up to 2% Tween 20.^41,42^ In our study, it improved the formation of dispersal colonies on the LB plate. Next, 100 μL of appropriate dilutions were plated onto agar-based media to ensure that the serial dilutions would give at least one countable plate (30–300 countable colonies per plate). Then, the plates were incubated at 37 °C to enumerate the colonies.

### Kill Curves

*E. coli* cells were grown overnight to the mid log phase (OD_600_ of 0.5–1) and diluted to OD 0.05, corresponding to ∼10^7^ CFU/ml in fresh LB medium. The concentrations of rifampicin used in the CFU assays were 4, 16, and 32 μg/mL. Next, CFU assays were performed, and the plates were incubated at 37 °C for 4 days until the colonies were enumerated.

### Minimum Inhibitory Concentration (MIC)

*E. coli* K-12 strain was grown in 5 mL of LB broth for 20 h at 37 °C with constant shaking at 200 rpm. Next, the cell culture was diluted in 10^6^ CFU/mL into a fresh LB medium as a working inoculum in a 15 mL tube. Then, 50 μL of culture was streaked over an LB agar surface, including rifampicin with the concentrations of 4, 16, and 32 μg/mL. Plates were incubated in an incubator overnight at 37 °C for 20 h. The MIC value was determined as the lowest concentration of antibiotic that inhibits the visible growth of bacteria. We confirmed the MIC value with three independent experiments.

### Mathematical Model

**Figure 1:**
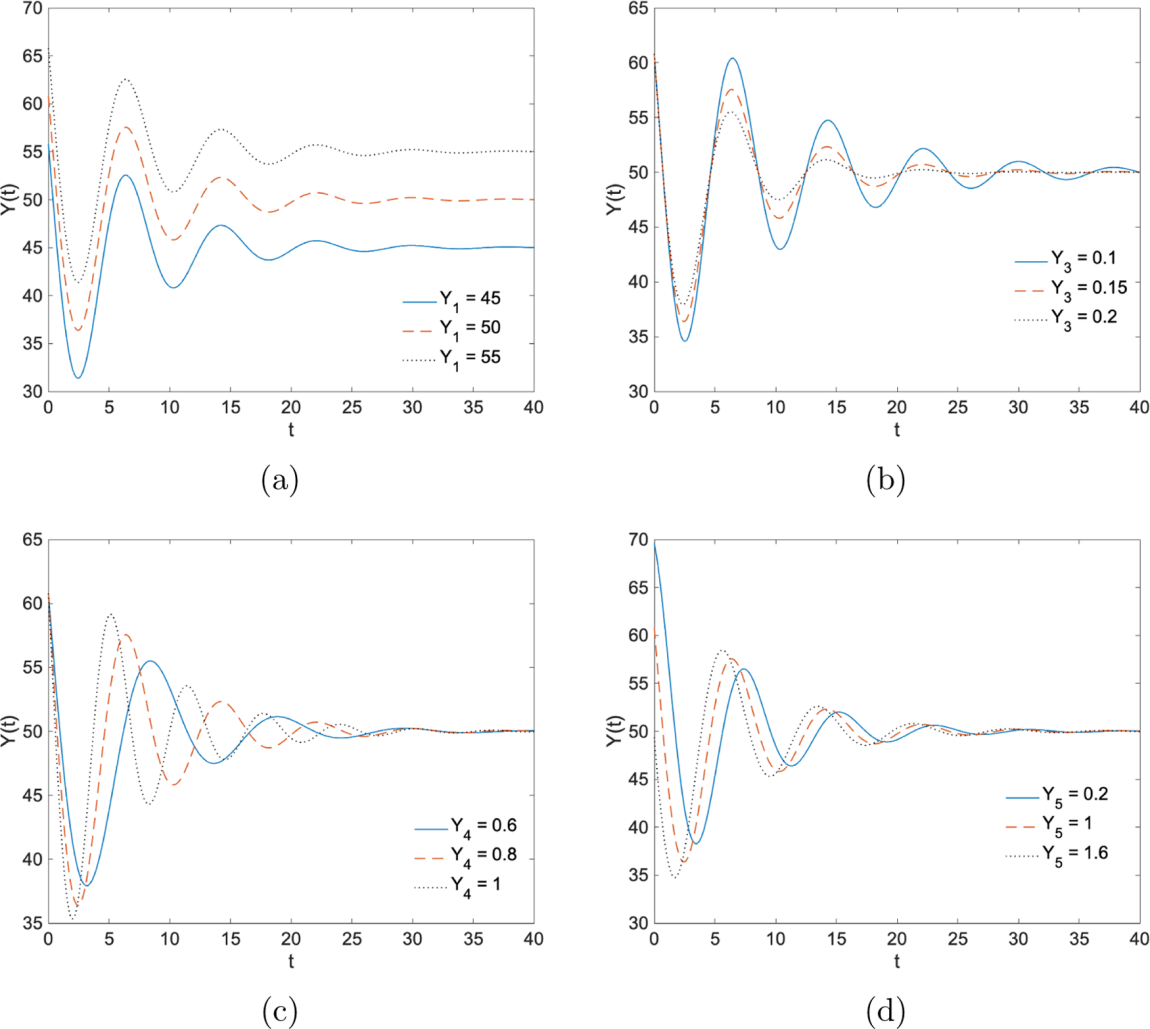
Illustration of the effects of the parameters in the proposed model. (a) *Y*_1_, (b) *Y*_3_, (c) *Y*_4_, and (d) *Y*_5_ is varied, while other parameters were at their default values; *Y*_1_ = 50, *Y*_2_ = 20, *Y*_3_ = 0.15, *Y*_4_ = 0.8, and *Y*_5_ = 1. *Y*(*t*) is plotted against time (*t*).

Damped oscillations are commonly observed in various natural and engineered systems, including mechanical systems, electrical circuits, and economics. In the literature, damped oscillations refer to repetitive, back-and-forth motions or vibrations in a system that gradually decrease in amplitude over time due to energy dissipation. In other words, the oscillations gradually lose energy, causing the system to come to its resting position. A similar oscillation with decreasing amplitude over time is also observed in the growth and killing kinetics of rifampicin-exposed *E. coli* cells. Damped oscillations can be mathematically represented using various equations depending on the properties and specifications of a system. One common way to represent damped oscillations is using a second-order linear differential equation. Hence, its solution provides the mathematical expression for the damped oscillations over time. To define the relationship between the rifampicin concentrations and the growth and killing kinetics of *E. coli* cells, we proposed to use the equation presented in (eq 1)
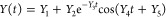
1Here, *Y*_1_, *Y*_2_, *Y*_3_, *Y*_4_, and *Y*_5_ are the model parameters that will be estimated. The parameter *Y*_1_ corresponds to the final value of *Y*(*t*) after diminishing all oscillations, *Y*_2_ shows the initial amplitude of the oscillations, *Y*_3_ accounts for the damping of oscillations, and *Y*_4_ and *Y*_5_ correspond to the frequency and phase shift of the oscillations, respectively. Figure 1 shows the effect of different parameter settings on the function *Y*(*t*). Figure 1a shows *Y*(*t*) for three different values of *Y*_1_ while other parameters were at their default values; *Y*_2_ = 20, *Y*_3_ = 0.15, *Y*_4_ = 0.8, and *Y*_5_ = 1. Each curve in Figure 1a shows oscillation, while the amplitude decreases exponentially and eventually reaches its equilibrium point (final value); *Y*_1_. Figure 1b shows the effect of the damping coefficient, *Y*_3_, on function *Y*(*t*). As *Y*_3_ increases, the amplitude of oscillations decreases much faster. The frequency and phase shift of the oscillations depend on the parameters *Y*_4_ and *Y*_5_, respectively. Figure 1c,1d shows the period and phase changes in *Y*(*t*) for different values of *Y*_4_ and *Y*_5_.

A nonlinear least-squares algorithm was used to obtain model parameters. Mathematically, the parameter vector θ = [*Y*_1_, *Y*_2_, *Y*_3_, *Y*_4_, *Y*_5_] was obtained by solving (eq 2)
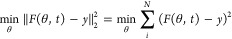
2Here, *t* is the given time, *y* is the measured normalized CFU data, *F*(θ, *t*) is the nonlinear model function given in (eq 1), and *N* is the total number of sample points. The implementation was performed in MATLAB R2020a computing environment by using lsqcurvefit function.^43^

## Results

**Figure 2:**
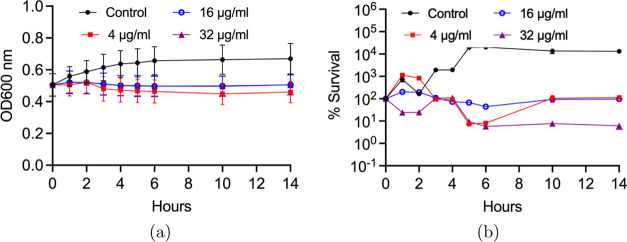
Optical density measurements and percentage of survival of *E. coli* cells. (a) Optical density measurements at the wavelength of 600 nm. (b) CFU killing assay of *E. coli* culture in the absence and presence of rifampicin 4, 16, and 32 μg/mL for 14 h. The symbols represent the average values, and the vertical bars at each data point indicate the standard deviations obtained from three separate experiments.

We performed optical density measurements to obtain growth kinetics of *E. coli* cells in the absence and presence of rifampicin, as detailed in the Methodology Section. The MIC value of *E. coli* cells exposed to rifampicin was 0–12 μg/mL as reported in ref (35). Figure 2a illustrates the growth kinetics of *E. coli* cells for the control and 4, 16, and 32 μg/mL rifampicin exposures.

To obtain the killing kinetics of *E. coli* cells, we performed a rifampicin killing assay as previously explained in the Methodology Section. We determined the number of colonies in the absence (control) and presence of a set of rifampicin doses for 14 h, as explained in the Methodology Section. Figure 2b shows the oscillatory behavior of *E. coli* cells in the early phase of the rifampicin treatment. As discussed in the Mathematical Model Section, we used (eq 1) to model this oscillatory behavior of *E. coli* cells. The results of the parameter search for the proposed model for the different concentrations of rifampicin are given in Table 1.

**Table 1:** Estimated Parameters of the Proposed Model (eq 1) for the Growth and Rifampicin Killing Kinetics of *E. coli* Cells

	0	4 μg/mL	16 μg/mL	32 μg/mL
*Y*_1_	13612	97	85.96	61.9
*Y*_2_	20038	2595.2	207.5	49.63
*Y*_3_	0.13	0.7	0.35	–0.08
*Y*_4_	0.54	–0.9	–0.84	–1.6
*Y*_5_	2.13	1.6	1.49	2.46

**Figure 3:**
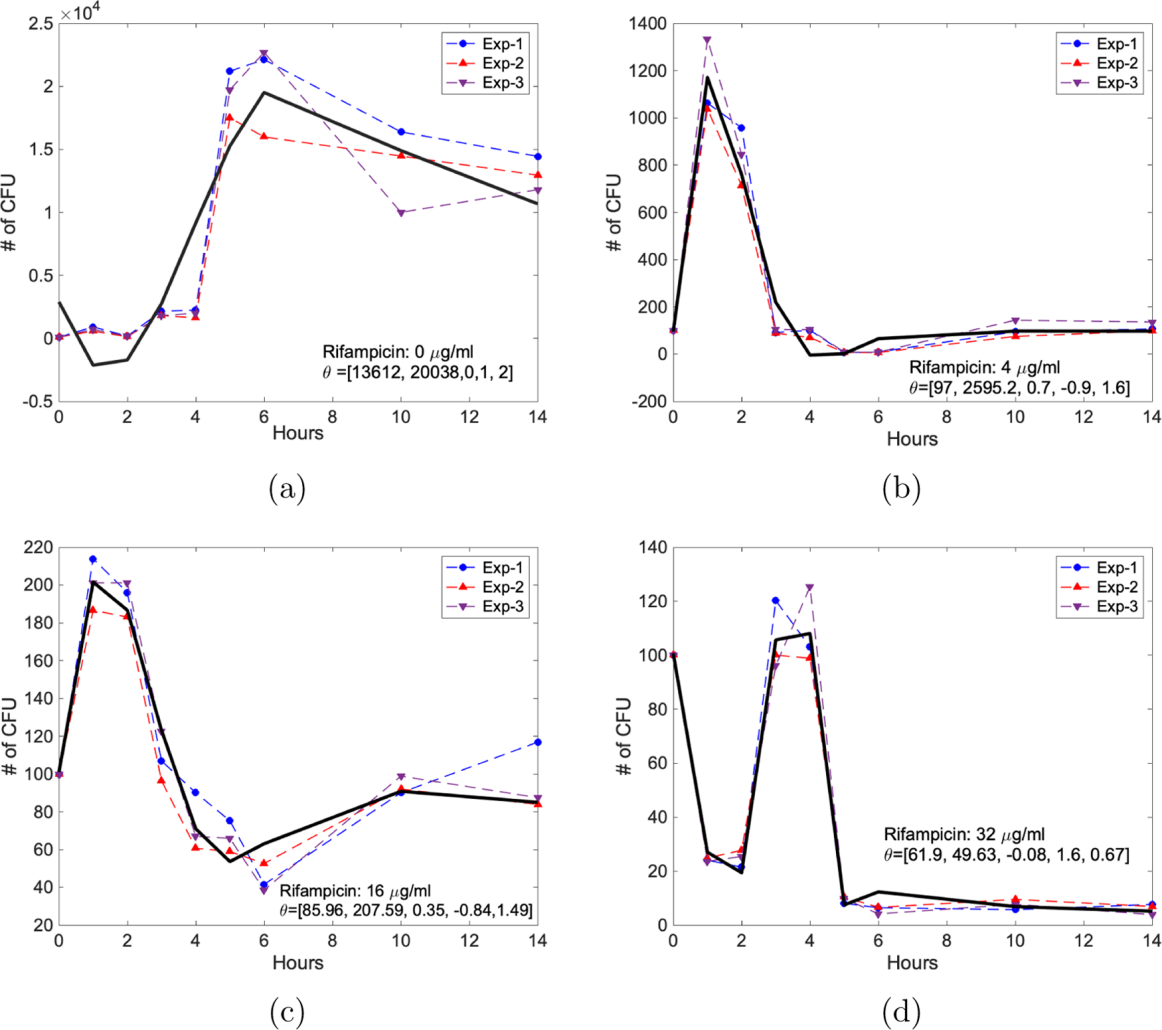
Curve fitting results for the response of *E. coli* cells to rifampicin: (a) in the absence of rifampicin, (b) 4 μg/mL, (c) 16 μg/mL, (d) 32 μg/mL rifampicin concentrations. The bacterial counts are plotted against time (hours). At time zero, the number of CFU is 100 under conditions (a–d). The model parameters are listed as θ = [*Y*_1_, *Y*_2_, *Y*_3_, *Y*_4_, *Y*_5_ ]. The solid black line shows the fitted curve of the model. Exp, the abbreviation for experiment. Three independent experiments were performed.

Figure 3 presents the curve fitting results for 0 μg/mL (Figure 3a), 4 μg/mL (Figure 3b), 16 μg/mL (Figure 3c), and 32 μg/mL (Figure 3d) rifampicin treatment of *E. coli* cells with the three independent experimental data.

**Figure 4:**
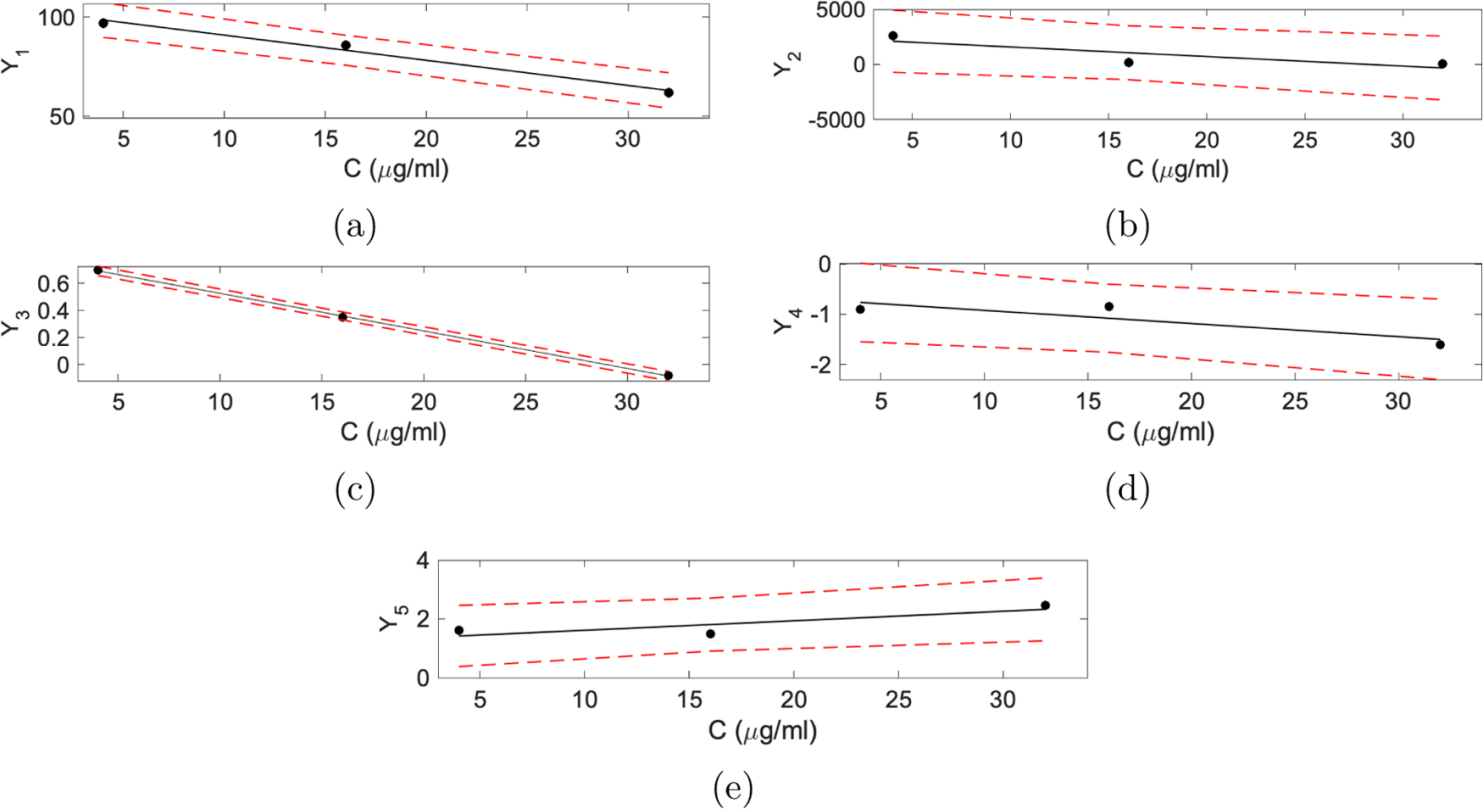
First-order polynomial fit for the model parameters. Change of (a) *Y_1_*, (b) *Y_2_*, (c) *Y_3_*, (d) *Y_4_*, and (e) *Y_5_* values according to rifampicin concentrations. The black dots represent the sample points; the black solid line depicts first-order polynomial fit, and the red dashed lines show the 95% prediction intervals.

The obtained first-order polynomial fit that presents the linear relationship between the model parameters and the concentration levels of rifampicin with a 95% prediction interval is shown in Figure 4. The corresponding linear relationships between the concentration level of rifampicin (*C*, μg/mL) and the model parameters are given in (eqs 3–7).
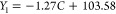
3
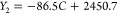
4
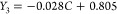
5
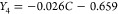
6
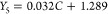
7

## Discussion

Rifampicin is a broad-spectrum antibiotic against various bacterial pathogens.^14−21^ It has a rapid killing rate in the first 6 h of treatment. The efficacy of rifampicin decreases over time due to the appearance of rifampicin persister or resistant phenotypes in the population.^44−46^ To benefit more from rifampicin, its current standard dosage might be reconsidered and deeply explored by using both computational tools and experimental or clinical studies. Our study can only give rise to various questions about how the initial killing kinetics of rifampicin influence its killing profile and, using mathematical models, whether we can predict its dose-dependent killing pattern. We believe that an improved standard dosage of rifampicin will increase cure rates, lower relapse rates, and, as a consequence, decrease mortality rates of several infectious diseases.^23,34^ In the literature, most of the mathematical models have been focused on modeling steady state antibiotic killing patterns of antimicrobials and using exponentially changing functions in modeling. Most of these models rely on conventional CFU data with a large sampling time (time points every 24 h) that is inadequate to observe the oscillatory behavior of antibiotic-treated bacteria.^34^ Here, our focus was to enhance the mathematical models to reveal better the response of *E. coli* cells to rifampicin exposure in the early phase. The initial growth of bacteria in the presence of rifampicin might contribute to the appearance of antibiotic-resistant cells or relapse of cells upon antibiotic treatment. Here, we obtained growth parameters of *E. coli* cells under regular growth conditions (control, without antibiotics) and in the presence of rifampicin exposure for 4, 16, and 32 μg/mL. In our experiments, the killing profile of rifampicin was biphasic; initially, cells grew more than being killed. Thereafter, rapid killing was followed by the growth of the cells in the presence of rifampicin, Figure 2. The MIC value of rifampicin for *E. coli* was reported as 0–12 μg/mL in the literature.^34^ First, we performed OD measurements of *E. coli* cells in the absence and presence of 4, 16, and 32 μg/mL concentrations of rifampicin via 2 h of sampling time for 14 h. We obtained a consistent biphasic profile of rifampicin killing in Figure 2a. To confirm, we performed a CFU assay using 4, 16, and 32 μg/mL concentrations of rifampicin, Figure 2b. Contrary to OD measurements, we obtained regrowth of *E. coli* cells in the presence of 4 μg/mL rifampicin after 6 h. Besides, the decline of CFU at a 32 μg/mL concentration of rifampicin was higher. Since bacteria debris might be optically detected and contribute to density measurements of the cells, we used the data generated by the CFU assay for the mathematical modeling. We applied the nonlinear least-squares algorithm in MATLAB to obtain the model parameters. We generated the mathematical model in (eq 1) with the listed model parameters in Table 1. Figure 3 demonstrates the numerical and experimental data of the growth and killing kinetics when we simulate rifampicin exposure. Figure 4 displays the first-order polynomial fit for these models with 95% prediction intervals. Figure 1 shows that the oscillatory behavior of the rifampicin-treated *E. coli* population can be described using (eq 1). The results presented here might raise more questions about the rifampicin killing profile, such as the underlying reasons for the increased and then decreased colony counts in the initial period of rifampicin treatment. Mostly, the sampling time of CFU assays in the literature is inadequate to exhibit the oscillatory behavior of antibiotic killing phase, which might contribute to the confer of antibiotic resistance.

## Conclusions

Kinetics of antimicrobial actions are generally used to evaluate and compare new drugs and study the differences and changes in the antimicrobial susceptibilities of bacterial populations. In our experiments, the killing profile of rifampicin was biphasic: initially, cells were growing more than being killed. Subsequently, rapid killing was followed by the growth of cells in the presence of rifampicin. To model the killing behavior of the cells at various rifampicin concentrations, we proposed to use the damped harmonic oscillator’s equation of motion and obtained model parameters by applying a nonlinear curve fitting solver in the MATLAB computing environment. The proposed mathematical model presents high fitting accuracy between the numerical results and time-killing curve data of *E. coli* cells for rifampicin exposure.

Our future work aims to enhance mathematical models for a unified description of *E. coli* cell survival with varying rifampicin concentrations, followed by the development of an open-source modeling library predicting dose-dependent antibiotic killing across diverse bacterial species for different types of antibiotics.
